# A 10-year observational study on the trends and determinants of smoking status

**DOI:** 10.1371/journal.pone.0200010

**Published:** 2018-07-06

**Authors:** Daryoush Samim, Marie Méan, Carole Clair, Pedro Marques-Vidal

**Affiliations:** 1 Department of medicine, internal medicine, Lausanne University Hospital, Lausanne, Switzerland; 2 Department of Ambulatory Care and Community Medicine, Policlinique Médicale Universitaire, Lausanne, Switzerland; Legacy, Schroeder Institute for Tobacco Research and Policy Studies, UNITED STATES

## Abstract

**Introduction:**

Most studies on motivation and intention to quit smoking have been conducted among adolescents and young adults but little is known regarding middle-aged subjects. We aimed to assess the trends and determinants of smoking status in a population-based cohort.

**Method:**

Observational, prospective study with a first mean follow-up at 5.6 years and a second at 10.9 years. Data from 3999 participants (49.2% women, aged 35–75 years) living in Lausanne (Switzerland).

**Results:**

Baseline prevalence of never, former and current smokers was 41.3, 34.3 and 24.3%, respectively. During the study period, more than 90% of never and former and almost 60% of current smokers at baseline retained their status after 10.9 years. Among 973 current smokers, 216 (22.2%) had quit for at least 5 years. Multivariable analysis showed increasing age to be positively associated with quitting (p-value for trend <0.001). Among 1373 former smokers, 149 (10.9%) had relapsed; increasing age (p-value for trend <0.001) was negatively associated and family history of lung disease was positively associated with relapse [OR and 95% CI: 1.53 (1.06–2.21)]. Among 1653 never smokers, 128 (7.7%) initiated smoking; Male gender [1.46 (1.01–2.12)] and living in coupled relationship [0.66 (0.45–0.97)] were associated with smoking initiation.

**Conclusion:**

Most middle-aged never and former smokers did not change their status with time, while 22.2% of current smokers sustained quitting. This is encouraging and could be improved with adequate supportive methods. In comparison to available data, this study confirms the difficult task of identifying subjects at risk of a negative behavioral change.

## Introduction

Cigarette smoking is the most important modifiable risk factor for premature death in the world, causing more than 6.4 million deaths yearly [1] and representing 5.7% of global health expenditure [2]. Prevalence of smoking varies between 8.7% to more than 35% in European countries with differing trends [3]. In Switzerland, smoking prevalence declined from 33% in 1997 to 28% in 2007, due to an increase in funding for tobacco control [4]. National data showed that, in 2015, 25% of Swiss adults smoked and that this prevalence tended to stabilize [5], but a recent study suggested that this prevalence might be underestimated [6].

A European study conducted in 2010 showed that older age, being divorced, having friends/family or parents who smoke were all significantly associated with ever smoking [7]. The influence of peers was also reported in Italy [8] and in a larger European study [9]. Interestingly, the last study found no association between design and marketing features of tobacco products and early initiation of regular smoking [9]. We have previously shown that over two third of Swiss smokers want to quit, but that only a small part wishes to do so in the short term [10]. Further, a study using nationally representative Swiss data showed an educational and income gradient in successful quitting and abstinence duration in favor of the people with higher socio-economic status [11].

Importantly, in these last studies, other sociodemographic covariates (place of birth and age of youngest child for example), clinical covariates, lifestyle, psychiatric disorders and use of smoking cessation aids use were not analysed. Also, most studies assessing the determinants of smoking initiation and cessation were either cross-section or conducted among adolescents and young adults [12], and information is lacking regarding middle-aged subjects using prospective data. Therefore, we aimed to assess the trends and determinants of smoking status in a middle aged, population-based cohort followed for a 10.9-year period.

## Population and methods

### Study design

The CoLaus Study (www.colaus.ch) aims to assess the prevalence of cardiovascular risk factors and to identify new molecular determinants of these risk factors in participants aged 35–75 years living in the city of Lausanne (Switzerland). The sampling procedure of the CoLaus Study has been described previously [13]. Briefly, the source population was defined as all subjects aged between 35 and 75 years of the population register of the city. All subjects living in Lausanne for more than 90 days have their names included in the register, which also includes information on age and sex. A simple, non-stratified random sample of 19,830 subjects (corresponding to 35% of the source population) was drawn and the selected subjects were invited by letter. If no answer was obtained, a second letter was sent, and if no answer was obtained, the subjects were contacted by phone. The baseline study began in June 2003 and ended in May 2006. The first follow-up was performed between April 2009 and September 2012, 5.6 years on average after baseline; The second follow-up was performed between May 2014 and July 2017, 10.9 years on average after baseline. All study periods included an interview, a physical exam and blood analysis.

### Smoking status

Smoking status was assessed at baseline, first and second follow-ups using self-reported data. Smoking status was defined as never, former (irrespective of the time since quitting) and current.

When a participant reported being a never smoker at one follow-up and had reported being current or former in the previous examination, the status was corrected to “former” (n = 37, 0.9%).

Participants who shifted their condition at first and/or second follow-up were secondly categorized into three subgroups: initiators if they were never smokers before and reported starting smoking; relapsers if they reported restarting smoking; quitters if they were current smokers at baseline if they reported quitting smoking at FU1 and FU2 (i.e. category CFF).

### Covariates

Socio-demographic and clinical covariates were collected by self-filled questionnaires, interview and/or physical examination. Educational level was categorized as low (obligatory school or apprenticeship), medium (high school), or high (university degree). Marital status was categorized into living as a couple or alone. Nationality was defined as Swiss born and other. Personal and family history of cardiovascular or pulmonary events were considered as present if the participant responded positively to the questions “have you or a member of your family (parents, siblings or children) been told that you had a cardiovascular disease (angina, myocardial infarction, stroke)” and “have you or a member of your family (parents, siblings or children) been told that you had lung disease (asthma, emphysema or chronic bronchitis)”

We defined participants as physically active if they exercised at least 20 minutes of leisure time physical activity per week [14, 15]. Alcohol consumption was self-reported and expressed in standard units consumed per week [16] or as alcohol drinkers (yes/no).

Personal history of anxiety and depression was collected by questionnaire (self-reported). In a subgroup of participants (n = 3719) aged between 35 and 66 years, current and previous occurrence of anxiety, depression and substance abuse were evaluated by a structured interview (PsyCoLaus study). Details of the methodology and characteristics of the PsyCoLaus sample have been previously described [17]. Briefly, the psychiatric baseline evaluations included the French version of the semi-structured Diagnostic Interview for Genetic Studies (DIGS) [18, 19]. In this study, we grouped all types of anxiety disorder and all types of depression. Substance abuse (ever or current) was defined as taking either THC, cocaine, amphetamines, solvents or opiates.

For participants with children (n = 3888), age of the youngest child was collected and categorized into <5 and ≥5 years. Our hypothesis was that parents with younger children would quit smoking to protect their children against the effects of second hand smoking.

Participants reported which medicines (prescribed or obtained over the counter) they consumed. Intake of smoking cessation aids, bupropion, varenicline and nicotine replacement therapy was obtained for baseline, first and second follow-ups. Due to differences in coding between surveys, the intake of varenicline and nicotine had to be grouped in a single variable.

Body weight and height were measured with participants standing without shoes in light indoor attire. Body weight was measured in kilograms to the nearest 100 g using a Seca^®^ scale (Hamburg, Germany). Height was measured to the nearest 5 mm using a Seca^®^ (Hamburg, Germany) height gauge. Blood pressure (BP) was measured using an Omron^®^ HEM-907 automated oscillometric sphygmomanometer after at least a 10-minute rest in a seated position, and the average of the last two measurements were used. Overweight was defined as 25≤ body mass index (BMI)<30 kg/m^2^ and obesity as BMI ≥30 kg/m^2^.

Blood samples were collected after an overnight fast and biological assays were performed by the Clinical Laboratory of the Lausanne university hospital on fresh blood samples within 2 hours of blood collection. All measurements were conducted in a Modular P apparatus (Roche Diagnostics, Switzerland). The following analytical procedures (with maximum inter and intra-batch CVs) were used: total cholesterol by CHOD-PAP (1.6%–1.7%); HDL-cholesterol by CHOD-PAP + PEG + cyclodextrin (3.6%–0.9%); triglycerides by GPO-PAP (2.9%–1.5%) and glucose by glucose dehydrogenase (2.1%–1.0%). LDL-cholesterol was calculated using the Friedewald formula.

Dyslipidemia was defined as an HDL-cholesterol <1 mmol/L in men and <1.29 mmol/L in women and/or LDL-cholesterol ≥4.1 mmol/L (≥2.6 mmol/L if personal history of CVD or diabetes) and/or triglyceride ≥2.2 mmol/L and/or presence of a hypolipidemic drug treatment. Hypertension was defined as a systolic BP ≥140 mm Hg and/or diastolic BP ≥90 mm Hg and/or presence of an antihypertensive drug treatment. Diabetes was defined as a fasting plasma glucose ≥7 mmol/L and/or presence of oral hypoglycaemic or insulin treatment.

### Exclusion criteria

We excluded patients who a) missed smoking data at baseline, first or second follow-up; b) did not participate in the follow-up (first or second) and c) missed covariates at baseline.

### Statistical analysis

Statistical analyses were performed using Stata version 15.0 for windows (Stata Corp, College Station, Texas, USA). Descriptive results were expressed as number of participants (percentage) for categorical variables and as average ± standard deviation or median and [interquartile range] for continuous variables. Bivariate analyses were performed using chi-square or Fisher’s exact test for qualitative variables and Student’s t-test, analysis of variance or Kruskall-Wallis test for quantitative variables.

Multivariable analysis was performed using stepwise logistic regression and the results were expressed as multivariable-adjusted odds ratio (OR) and 95% confidence interval (CI). Both forward and backward logistic regressions were performed to assess consistency of the results. All variables included in the bivariate analysis (i.e. sex, age, education, country of birth, marital status, BMI categories, personal and family history of cardiovascular and lung disease, hypertension, dyslipidaemia, diabetes, alcohol consumption, physical activity, anxiety and depression) were included in the stepwise process. Multivariable analysis were performed in two steps.

Firstly, we analysed factors associated with initiating, relapsing or quitting and maintaining quitting (i.e. category “CFF”) among never, former and current smokers, respectively. Second, sensitivity analyses were conducted as follows: 1) including age of the youngest child in the model and 2) using the psychological assessments performed in PsyCoLaus instead of the self-reported ones. The first sensitivity analysis was conducted because we hypothesized that parents with young children might change their smoking habits to prevent second-hand smoking. The second sensitivity analysis used objective, professionally diagnosed diseases instead of self-reported ones. Statistical significance was assessed for a two-sided test with p<0.05.

### Ethical statement

The institutional Ethics Committee of the University of Lausanne (Canton de Vaud, Commission cantonale d'éthique de la recherche sur l'être Humain; Available from: http://www.cer-vd.ch/2014) approved the baseline CoLaus study (protocol reference 16/03, decisions of 13^th^ January and 10^th^ February 2003) and the approval was renewed for first (protocol reference 33/09, decision of 23^rd^ February 2009) and second (reference 26/14, decision of 11^th^ March 2014) follow-ups. The full decisions can be obtained from authors upon request. The study was performed in agreement with the Helsinki declaration and in accordance with the applicable Swiss legislation. All participants gave their signed informed consent before entering the study.

## Results

### Characteristics of participants

**Fig 1:**
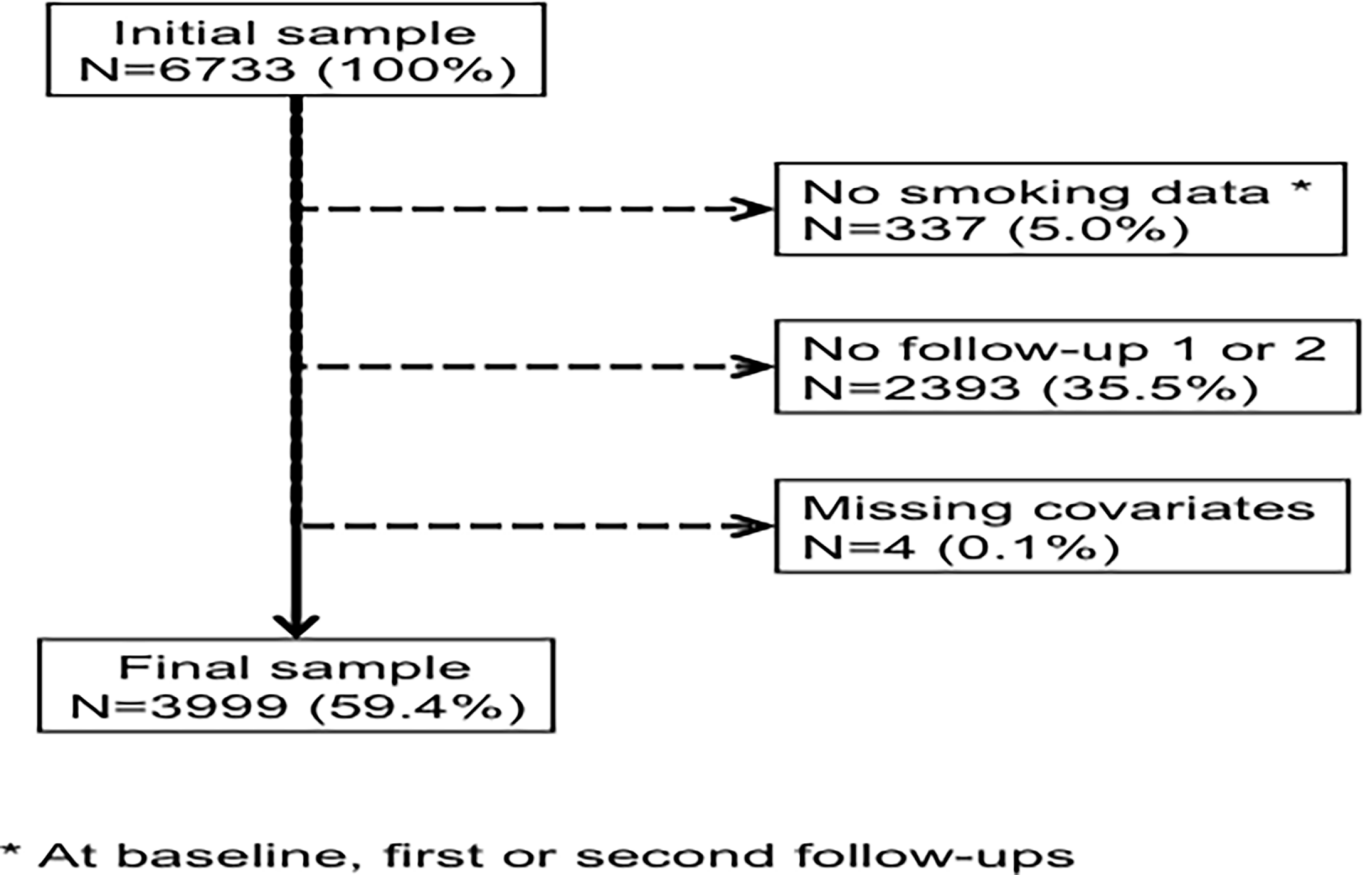
Number of participants excluded and retained for analysis.

Of the initial 6733 participants, 3999 (59.4%) were retained for analysis. The reasons for exclusion are indicated in **Fig 1**and the characteristics of included and excluded participants are summarized in S1 **Table**. Included participants were younger, more often women, with higher education level, living in coupled circumstance and born in Switzerland. They reported less anxiety and depression, were more engaged in a physical activity, had a lower BMI, lower levels of hypertension, dyslipidemia and diabetes, and less personal history of cardiovascular disease (CVD). Included participants also were more often alcohol drinkers and had more often a family history of lung disease. Finally, included participants were more frequently former smokers and less frequently current smokers.

The baseline characteristics of the included participants according to smoking status are summarized in **Table 1**.

**Table 1:** Baseline characteristics of never, former and current smokers, CoLaus study, Lausanne, Switzerland.

Baseline smoking status	Never	Former	Current	P-value
N	1653	1373	973	
Sex (male)	637 (38.5)	707 (51.2)	457 (47.0)	<0.001
Age categories				<0.001
[35–44]	557 (33.7)	340 (24.8)	347 (35.7)	
[45–54]	447 (27.0)	437 (31.8)	337 (34.6)	
[55–64]	441 (26.7)	399 (29.1)	221 (22.7)	
[65–75]	208 (12.6)	197 (14.3)	68 (7.0)	
Education level				<0.001
University	389 (23.5)	324 (23.6)	180 (18.5)	
High school	443 (26.8)	384 (28.0)	252 (25.9)	
Apprenticeship	557 (33.7)	507 (36.9)	368 (37.8)	
Primary level	264 (16.0)	158 (11.5)	173 (17.8)	
Born in Switzerland	1069 (64.7)	905 (65.9)	606 (62.3)	0.191
Marital status (in couple)	1136 (68.7)	989 (72.0)	609 (62.6)	<0.001
Personal history				
Cardiovascular disease	63 (3.8)	100 (7.3)	34 (3.5)	<0.001
Lung disease	178 (10.8)	164 (11.9)	124 (12.7)	0.287
Family history				
Cardiovascular disease	625 (37.8)	575 (41.9)	339 (34.8)	0.002
Lung disease	405 (24.5)	369 (26.9)	228 (23.4)	0.132
Hypertension	506 (30.6)	490 (35.7)	247 (25.4)	<0.001
Body mass index categories				<0.001
Normal	908 (54.9)	630 (45.9)	539 (55.4)	
Overweight	540 (32.7)	534 (38.9)	339 (34.8)	
Obese	205 (12.4)	209 (15.2)	95 (9.8)	
Dyslipidaemia	557 (33.7)	558 (40.7)	3 89 (40.0)	<0.001
Diabetes	58 (3.5)	90 (6.5)	40 (4.1)	<0.001
Alcohol drinker	120 (67.8)	1098 (80.0)	784 (80.6)	<0.001
Physically active	982 (59.4)	823 (59.9)	460 (47.3)	<0.001
Anxiety, self-reported	123 (7.4)	112 (8.2)	101 (10.4)	0.030
Depression, self-reported	220 (13.3)	232 (16.9)	191 (19.6)	<0.001
Sensitivity analyses				
Child aged <5 years	137 (14.1)	82 (10.2)	65 (12.5)	0.048
Diagnosed psychiatric diseases	729 (44.1)	790 (57.5)	698 (71.7)	
Substance use, ever	39 (2.4)	154 (11.2)	171 (17.6)	<0.001
Anxiety, ever	251 (15.2)	246 (17.9)	186 (19.1)	0.109
Depression, ever	439 (26.6)	390 (28.4)	341 (35.0)	0.006

Results are expressed as number of participants (percentage) for categorical data. Between-group comparisons using chi-square test.

### Trends in smoking categories

**Fig 2:**
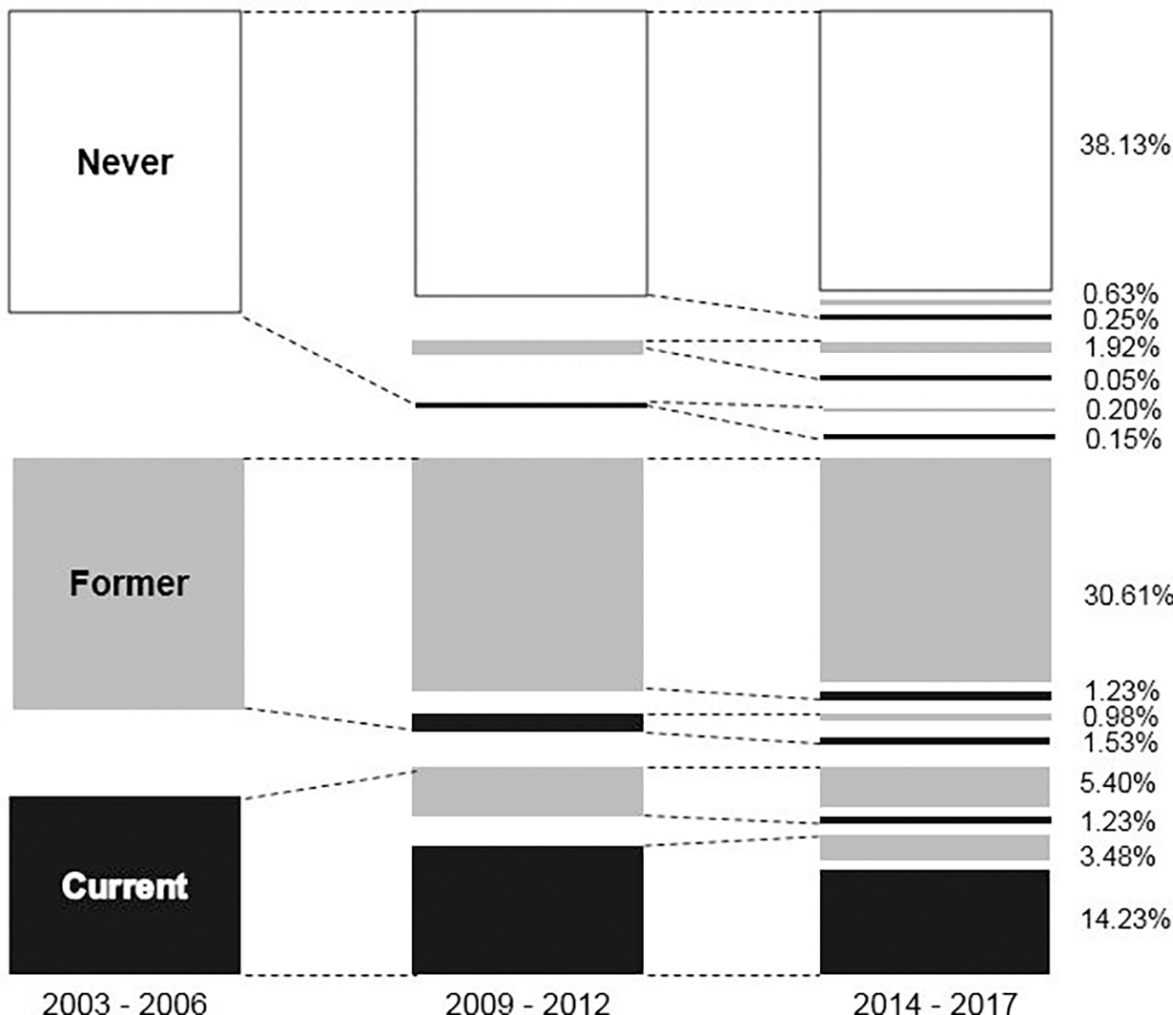
Smoking categories at baseline, first a second follow-up.

The different smoking categories at baseline, first and second follow-ups are summarized in **Fig 2**. Most participants did not change their smoking status during follow-up. Among all participants, almost 40% of participants remained never smokers, 30% former smokers and 14% current smokers. Approximately 36% of current smokers at baseline quitted smoking at FU1 and/or FU2. During the study period, more than 90% of never, 90% of former and almost 60% of current smokers at baseline retained their status after 10 years (**Fig 2**).

### Determinants of quitting smoking

Among the 973 current smokers, 216 (22.2%) quit and remained former smokers for at least 5 years. The bivariate analysis of the factors associated with quitting is summarized in **Table 2**. Increasing age had a higher frequency of quitting. This was further confirmed in both forward and backward multivariable analysis. Relative to the age group [35–44], the OR and 95% CI were 0.76 (0.51–1.13), 1.52 (1.01–2.30) and 2.76 (1.50–5.09) for age groups [45–54], [55–64] and [65–75], respectively (p-value for trend <0.001) (**S2 Table**). The first sensitivity analysis confirmed the association between age and quitting (p-value for trend <0.001) and identified children aged <5 years [0.41 (0.21–0.80)] and dyslipidemia [0.59 (0.37–0.93)] as being negatively associated with quitting. The second sensitivity analysis showed that relative to university level, the OR and 95% CI were 0.61 (0.36–1.06), 0.52 (0.31–0.88) and 0.56 (0.30–1.07) for high school, apprenticeship and basic school, respectively (p-value for trend = 0.064) (**S2 Table**).

**Table 2:** Bivariate analysis of the factors associated with initiation, relapse or quitting smoking, CoLaus study, Lausanne, Switzerland.

	Initiators §			Relapsers ‡			Quitters †		
	No	Yes	p-value	No	Yes	p-value	No	Yes	p-value
N	1525	128		1224	149		569	216	
Sex (male)	579 (38.0)	58 (45.3)	0.101	631 (51.6)	76 (51.0)	0.900	255 (44.8)	105 (48.6)	0.341
Age categories			0.943			<0.001			<0.001
[35–44]	516 (33.8)	41 (32.0)		280 (22.9)	60 (40.3)		201 (35.3)	71 (32.9)	
[45–54]	410 (26.9)	37 (28.9)		386 (31.5)	51 (34.2)		219 (38.5)	57 (26.4)	
[55–64]	408 (26.8)	33 (25.8)		369 (30.2)	30 (20.1)		122 (21.4)	63 (29.2)	
[65–75]	191 (12.5)	17 (13.3)		189 (15.4)	8 (5.4)		27 (4.8)	25 (11.6)	
Education level			0.350			0.123			0.082
University	360 (23.6)	29 (22.7)		280 (22.9)	44 (29.5)		94 (16.5)	53 (24.5)	
High school	408 (26.8)	35 (27.3)		338 (27.6)	46 (30.9)		155 (27.2)	51 (23.6)	
Apprenticeship	520 (34.1)	37 (28.9)		463 (37.8)	44 (29.5)		221 (38.8)	77 (35.6)	
Primary	237 (15.5)	27 (21.1)		143 (11.7)	15 (10.1)		99 (17.4)	35 (16.2)	
Born in Switzerland	993 (65.1)	76 (59.4)	0.192	813 (66.4)	92 (61.7)	0.255	373 (65.6)	128 (59.3)	0.101
Marital status (in couple)	1057 (69.3)	79 (61.7)	0.075	878 (71.7)	111 (74.5)	0.478	340 (59.8)	137 (63.4)	0.347
Personal history									
Cardiovascular disease	57 (3.7)	6 (4.7)	0.590	92 (7.5)	8 (5.4)	0.341	19 (3.3)	9 (4.2)	0.577
Lung disease	165 (10.8)	13 (10.2)	0.816	146 (11.9)	18 (12.1)	0.957	66 (11.6)	32 (14.8)	0.224
Family history									
Cardiovascular disease	580 (38.0)	45 (35.2)	0.519	515 (42.1)	60 (40.3)	0.673	195 (34.3)	73 (33.8)	0.900
Lung disease	375 (24.6)	30 (23.4)	0.771	318 (26)	51 (34.2)	0.032	135 (23.7)	49 (22.7)	0.759
Hypertension	463 (30.4)	43 (33.6)	0.446	444 (36.3)	46 (30.9)	0.194	137 (24.1)	63 (29.2)	0.144
Body mass index categories			0.422			0.120			0.991
Normal	841 (55.2)	67 (52.3)		550 (44.9)	80 (53.7)		315 (55.4)	120 (55.6)	
Overweight	492 (32.3)	48 (37.5)		483 (39.5)	51 (34.2)		200 (35.2)	75 (34.7)	
Obese	192 (12.6)	13 (10.2)		191 (15.6)	18 (12.1)		54 (9.5)	21 (9.7)	
Dyslipidemia	512 (33.6)	45 (35.2)	0.716	507 (41.5)	51 (34.5)	0.102	238 (42.0)	83 (37.5)	0.367
Diabetes	54 (3.5)	4 (3.1)	0.806	83 (6.8)	7 (4.7)	0.332	27 (4.8)	9 (4.2)	0.729
Alcohol drinker	1035 (67.9)	85 (66.4)	0.734	983 (80.3)	115 (77.2)	0.368	450 (79.1)	173 (80.1)	0.756
Physically active	907 (59.5)	75 (58.6)	0.845	736 (60.1)	87 (58.4)	0.682	249 (43.8)	107 (49.5)	0.147
Anxiety, self-reported	111 (7.3)	12 (9.4)	0.385	97 (7.9)	15 (10.1)	0.367	60 (10.5)	21 (9.7)	0.735
Depression, self-reported	206 (13.5)	14 (10.9)	0.411	200 (16.3)	32 (21.5)	0.114	118 (20.7)	36 (16.7)	0.200
N ǂ	892	81		709	94		296	126	
Child aged <5 years ǂ	128 (14.4)	9 (11.1)	0.422	66 (9.3)	16 (17.0)	0.020	32 (10.8)	20 (15.9)	0.148
Diagnosed, N ¶	979	79		781	107		417	140	
Substance use, ever	31 (3.2)	8 (10.1)	0.002	132 (16.9)	22 (20.6)	0.348	104 (24.9)	27 (19.3)	0.172
Anxiety, ever	230 (23.5)	21 (26.6)	0.535	209 (26.8)	37 (34.6)	0.090	122 (29.3)	33 (23.6)	0.194
Depression, ever	414 (42.3)	25 (31.7)	0.065	336 (43)	54 (50.5)	0.146	196 (47.0)	74 (52.9)	0.230

§ among never smokers at baseline

‡ among former smokers at baseline

† among current smokers at baseline

ǂ, among participants with children

¶, among participants in the PsyCoLaus study.

Results are expressed as number of participants (percentage); between-group comparisons using chi-square.

### Determinants of relapsing smoking

Among the 1373 former smokers, 149 (10.9%) relapsed. The bivariate analysis of the factors associated with smoking relapse is summarized in **Table 2**. Increasing age had a lower frequency of relapse, and this was further confirmed in both forward and backward stepwise multivariable analysis: relative to the age group [35–44], the OR and 95% CI were 0.60 (0.40–0.90), 0.37 (0.23–0.59) and 0.20 (0.09–0.42) for age groups [45–54], [55–64] and [65–75], respectively (p-value for trend <0.001). The multivariable analysis also identified family history of lung disease as being positively associated with relapse: 1.53 (1.06–2.21) (**S2 Table**). The first sensitivity analysis confirmed age as being inversely associated with relapse in both the forward and the backward selection procedure (p-value for trend <0.001), while no effect was found for family history of lung disease. The second sensitivity analysis confirmed age (p-value for trend <0.001) and family history of lung disease [1.59 (1.03–2.46)] as being associated with relapse (**S2 Table**).

### Determinants of smoking initiation

Among the 1653 never smokers, 128 (7.7%) initiated smoking. The bivariate analysis of the factors associated with smoking initiation is summarized in **Table 2**. No clinical or lifestyle factor was associated with initiation; backward multivariable analysis identified male gender OR and 95% CI: 1.46 (1.01–2.12) and living in as a couple 0.66 (0.45–0.97) as being associated with smoking initiation, while no variable was found in the forward procedure (**S2 Table**). The first sensitivity analysis confirmed the association between gender and smoking initiation in both the forward and the backward selection procedure: 1.60 (1.02–2.53). The second sensitivity analysis showed substance abuse [3.70 (1.62–8.42)] and depression [0.60 (0.37–0.99)] to be associated with initiation, while the effect of male gender was no longer significant (**S2 Table**).

## Discussion

Few studies have assessed the determinants of smoking initiation and cessation among middle-aged subjects. Using 10-year prospective data, we show that, among people aged 35 to 75 years, the majority of never and former smokers retain their status, while four out of ten smokers will make at least one quit attempt and one fifth will quit and maintain this status for at least 5 years. Our results also show that quitting is positively associated with older age and negatively associated with having children aged <5 years or dyslipidaemia. Relapsing is positively associated with younger age and family history of lung disease, while initiation is positively associated with male gender, living alone and substance abuse, and negatively associated with depression.

### Smoking prevalence

Prevalence of current smokers at baseline was 24.3%, a value comparable to Swiss national data in 2015 (25% of Swiss adults) but lower than pooled data from the 2001–2007 editions of the Swiss Tobacco Monitoring Survey (30.9%) [11]. A first explanation is that the former study included participants aged 14–65 years-old, thus over 10-years younger than our participants (35–75 years-old). A second explanation is the fact that our study was a prospective one, and it is well known that smokers tend to quit more frequently [20]; hence, it is likely that the prevalence of current smokers in our study is underestimated.

### Determinants of quitting smoking

Approximately one in five current smokers sustained quitting, a value considerably higher than reported in an Italian study (6.9%) [21]. A possible explanation is that the latter study was partly based on data derived from periods during which funding for tobacco control and public health measures were limited. Another possible explanation is that the Italian study included participants aged 25–64 years-old, thus 10-years younger than our participants (35–75 years-old).

Indeed, increasing age was positively associated with quitting smoking. This finding is in agreement with findings from Marti who showed that older smokers are more likely to quit successfully compared with smokers aged 18–24 years [11]. It is also in agreement with the previous finding that increasing age is negatively associated with the risk of relapsing. A likely explanation is that former smokers quit because of current tobacco-related health conditions and because of the future harmful effect of smoking on health [22]. As older smokers have more tobacco-related health conditions and more comorbidities, they are motivated to quit more often.

Dyslipidaemia was negatively associated with quitting smoking. Our finding partly disagrees with a previous cohort study [23] which showed that newly diagnosed dyslipidaemia was positively associated with quitting. However, the positive association was only significant for men and no association was found for known dyslipidaemia. Furthermore, the latter study was based on subjects aged 40 years at baseline. A possible explanation is that a significant fraction of dyslipidaemic subjects are not aware of their status [24, 25] and thus do not take preventive measures to reduce their CV risk factor levels.

Lower educational level tended to be negatively associated with quitting in the second sensitivity analysis. This is in agreement with a previous study conducted in Switzerland showing a positive educational and income gradient in successful cessation and abstinence duration for both genders [11]. The reasons for lower educated subjects to be less prone to quit smoking are varied [26]. Lower educated subjects have a lower health literacy, making them less receptive to preventive messages [27]. Lower health literacy is also associated with more positive smoking outcome expectancies (e.g., smoking facilitates social interactions, smoking reduces boredom or negative affect) and less negative smoking outcome expectancies (e.g., smoking is harmful to health) [26].

Having a child aged <5 years was negatively associated with quitting smoking. This unexpected finding is in disagreement with a previous cross-sectional study [28] which showed that parents with a child under age 3 years had higher odds of successfully quitting at 12 months. One possible explanation for our finding is that participants with children aged <5 years were younger (41.3±5.5 vs. 54.1±9.6 years, p<0.001), and that increasing age was positively associated with quitting smoking.

### Determinants of smoking relapse

Approximately 11% of former smokers relapsed during follow-up. This value is lower than the relapse rate reported in a workplace study conducted in Switzerland (14.4% after intervention and 31.1% at one year) [29]. Still, as relapse rates are dependent of several factors such as smoking dependency or time after quitting, comparison with other studies is difficult, as those variables were not collected in our study.

Increasing age was negatively associated with smoking relapse, a finding in agreement with a previous study [30]. Our results suggest that as age increases, likelihood of relapsing decreases, possibly due to the occurrence of tobacco-(un)related diseases and the subsequent increased pressure from health professionals to stop smoking.

Family history of lung disease was positively associated with smoking relapse. To our knowledge, this is the first study assessing the association between personal or family history of lung disease and smoking relapse. Previous studies showed that having parents who smoke was significantly associated with smoking [7–9]. Thus, participants with family history of lung disease may be more prone to relapse because of a more unfavorable family environment regarding smoking.

### Determinants of smoking initiation

In our study, 7.7% never smokers initiated smoking in this older group, a value considerably higher than reported in New Zealand (<1.0%) [31]. A possible explanation is the fact that Switzerland has only a partial smoking ban and further restrictions have been rejected by the population [32]. Switzerland is one of the few European countries which has signed but not ratified The Framework Convention on Tobacco Control [33]. There is therefore a permissive politic with relatively low prices for tobacco, few restrictions on marketing in particular indirect marketing and easy access to tobacco products. We postulate that the rather benevolent legislation regarding smoking in Switzerland might favour smoking initiation even among middle-aged subjects.

Male gender was positively associated with smoking initiation. This finding is in agreement with several cohort studies conducted among North American adults and adolescents [34–37], but not with a recent cohort study conducted among young Canadian adults [38]. Possible explanations include a lower health awareness among men and the fact that men still consider smoking as a masculine characteristic, although a better assessment of the rationale for middle-aged men to initiate smoking is needed.

Living as a couple was negatively associated with smoking initiation. This finding is in agreement with two cohort studies [34, 39] and another Swiss [11] and European [7] cross-sectional studies. A possible explanation is the mutual psychological support and influence, which might prevent smoking initiation.

Substance abuse was strongly associated with smoking initiation. This finding is in agreement with a systematic review [37] which showed that the use of alcohol and illegal drugs was associated with smoking initiation. Similarly, a cohort study [34] among American adults confirmed that substance use disorder is associated with smoking initiation. Possible explanations include a genetic predisposition regarding joint consumption of tobacco and other substances, as well as environmental conditions favouring multisubstance use [40].

No association was found between self-reported depression and smoking initiation in the initial analyses, while on sensitivity analysis a negative association between having been ever depressed and smoking initiation was found. This latter finding disagrees with other studies, which have shown a positive association between depression and smoking initiation [34, 41–43]. Several explanations can be put forward to explain these contrasting results. Firstly, most studies focused on adolescents [41–43], and the reasons for smoking initiation in adolescents might differ from those among middle-aged adults [44]. Secondly, depression was grouped with all axis I clinical disorders in one study [34] and based on self-reported symptoms in others [41–43], while in our sensitivity analysis it was objectively diagnosed using validated criteria. Indeed, a possible explanation for the negative association between depression and smoking initiation is that depressed subjects are advised by their doctors not to use smoking as a deterrent for their depressive symptoms. Still, the association between depression and smoking initiation needs further investigation.

### Strengths and limitations

To our knowledge, this is the first study exploring demographic, clinical, psychiatric and lifestyle determinants of smoking changes in a population-based sample.

This study has also some limitations. Firstly, participants excluded from the analysis were more frequently smokers. Hence, it is likely that the smokers included in the analysis were more health conscious and thus more prone to quit; our quitting rates might be overestimated. Secondly, despite collecting smoking cessation aids (bupropion and varenicline/nicotine), we could not include them in our analysis for different reasons. Indeed, Bupropion was also prescribed as an antidepressant for never smokers and current smokers. Therefore, we could not analyse its association with quitting smoking. On the other hand, despite being the most commonly (16.2%) used smoking cessation aid in Switzerland in 2013(41), six participants only reported using nicotinic replacement. In spite of asking for prescribed or obtained over the counter medicines in our questionnaires, participants may have probably under-reported nicotinic replacement. Thirdly, it was not possible to assess duration of smoking/quitting, or the magnitude of smoking dependency at baseline. Hence, it was not possible to assess whether tobacco-related factors influenced smoking trajectories, although it has been shown that increased tobacco dependency negatively influences quitting [45]. Fourthly, smoking status was assessed during the baseline and follow-up visits, and we have no information regarding smoking status between visits. Hence, a participant might be considered as a quitter in both follow-ups while he/she actually relapsed and quit again between visits. Hence, our quitting rates might be overestimated. Still, our results show that one fifth of current smokers will quit over 10 years, which is encouraging and could be even improved if adequate supportive methods were provided.

### Conclusion

In this population-based prospective study, most middle-aged never and former smokers do not change their status with time, while one fifth of current smokers will quit permanently. The determinants of change vary according to the smoking status. In comparison to available data, this study confirms the difficult task to identify subjects at risk of negative behaviour change.

## Supporting information

S1 Table Baseline socio-demographic and clinical characteristics of included and excluded participants. (DOCX)

S2 Table Multivariable analysis of the factors associated with initiation, relapse or quitting smoking according to selection procedure. (DOCX)
