# Supplementary material for: A 10-year observational study on the trends and determinants of smoking status
Source: PLoS One. 2018 Jul 6;13(7):e0200010. doi: 10.1371/journal.pone.0200010 (PMC6034816; doi:10.1371/journal.pone.0200010)
Supplement: S1 Table — (DOCX) [file pone.0200010.s001.docx]

**S1 Supporting information**

**S1 Table**: baseline socio-demographic and clinical characteristics of included and excluded participants

|  | **Included** | **Excluded** | **P-value** |
| --- | --- | --- | --- |
| N | 3999 | 2734 |  |
| Age (years) | 51.8 ± 10.3 | 53.8 ± 11.2 | <0.001 |
| Man (%) | 1801 (45.0) | 1388 (55.8) | <0.001 |
| Education level (%) |  |  | <0.001 |
| University | 893 (22.3) | 427 (15.7) |  |
| High school | 1079 (27.0) | 546 (20.0) |  |
| Apprenticeship | 1432 (35.8) | 945 (34.7) |  |
| Mandatory | 595 (14.9) | 802 (29.5) |  |
| Living alone (%) | 1265 (31.6) | 949 (34.8) | 0.007 |
| Born in Switzerland (%) | 2580 (64.5) | 1451 (53.1) | <0.001 |
| Age of the youngest child (years) | 19.5 ± 12.3 | 21.4 ± 13.4 | <0.001 |
| Personal history of |  |  |  |
| Cardiovascular disease (%) | 197 (4.9) | 210 (7.7) | <0.001 |
| Lung disease (%) | 466 (11.7) | 344 (12.6) | 0.250 |
| Family history of |  |  |  |
| Cardiovascular disease (%) | 1539 (38.5) | 1054 (38.6) | 0.956 |
| Lung disease (%) | 1002 (25.1) | 592 (21.7) | 0.001 |
| Hypertension (%) | 1243 (31.1) | 1149 (42.0) | <0.001 |
| Systolic BP (mmHg) | 127 ± 17 | 130 ± 18 | <0.001 |
| Diastolic BP (mmHg) | 79 ± 11 | 80 ± 11 | <0.001 |
| Body mass index (kg/m^2^) | 25.4 ± 4.3 | 26.4 ± 4.8 | <0.001 |
| Body mass index categories (%) |  |  | <0.001 |
| Normal | 2077 (51.9) | 1163 (42.5) |  |
| Overweight | 1413 (35.3) | 1049 (38.4) |  |
| Obese | 509 (12.7) | 522 (19.1) |  |
| Dyslipidemia (%) | 1504 (37.6) | 1280 (47.0) | <0.001 |
| Diabetes (%) | 188 (4.7) | 248 (9.1) | <0.001 |
| Smoking categories (%) |  |  | <0.001 |
| Never | 1653 (41.3) | 1079 (39.6) |  |
| Former | 1373 (34.3) | 810 (29.7) |  |
| Current | 973 (24.3) | 839 (30.8) |  |
| Alcohol drinkers (%) | 3002 (75.1) | 1816 (66.4) | <0.001 |
| Alcohol consumption (units/week) | 4 [1 - 9] | 3 [0 - 10] | §0.01 |
| Physical activity (%) | 2265 (56.6) | 1330 (48.8) | <0.001 |
| Anxiety (%) | 336 (8.4) | 303 (11.1) | <0.001 |
| Depression (%) | 643 (16.1) | 511 (18.7) | 0.005 |
| Medicines (%) |  |  |  |
| Bupropion | 66 (1.7) | 66 (2.4) | 0.026 |
| Varenicline/nicotine | 6 (0.2) | 1 (0.0) | 0.156 |

Results are expressed as number of participants (percentage) for categorical data, as average ± standard deviation or as median [interquartile range] for continuous variables. Between-group comparisons using chi-square for categorical variables and student’s test or Kruskal-Wallis test (§) for continuous variables
