# Supplementary material for: A 10-year observational study on the trends and determinants of smoking status
Source: PLoS One. 2018 Jul 6;13(7):e0200010. doi: 10.1371/journal.pone.0200010 (PMC6034816; doi:10.1371/journal.pone.0200010)
Supplement: S2 Table — (DOCX) [file pone.0200010.s002.docx]

**S2 Supporting information**

**S2 Table**: multivariable analysis of the factors associated with initiation, relapse or quitting smoking according to selection procedure, CoLaus study, Lausanne, Switzerland

|  | **Initiators** § | | **Relapsers** ‡ | **Quitters** † |
| --- | --- | --- | --- | --- |
| **Selection procedure** | **Backward** | **Forward** | **Backward and Forward** | **Backward and Forward** |
| Sex (man) | 1.46 (1.01-2.12) | - | - | - |
| Age categories | - | - | - | - |
| [35-44] | - | - | Ref. | Ref. |
| [45-54] | - | - | 0.60 (0.40-0.90) | 0.76 (0.51-1.13) |
| [55-64] | - | - | 0.37 (0.23-0.59) | 1.52 (1.01-2.30) |
| [65-75] | - | - | 0.20 (0.09-0.42) | 2.76 (1.50-5.09) |
| Education level | - | - | - | - |
| University | - | - | - | Ref. ¶ |
| High school | - | - | - | 0.61 (0.36-1.06) * |
| Apprenticeship | - | - | - | 0.52 (0.31-0.88) * |
| Mandatory | - | - | - | 0.56 (0.30-1.07) * |
| Born in Switzerland | - | - | - | - |
| Marital status (in couple) | 0.66 (0.45-0.97) | - | - | - |
| Personal history | - | - | - | - |
| Cardiovascular disease | - | - | - | - |
| Lung disease | - | - | - | - |
| Family history | - | - | - | - |
| Cardiovascular disease | - | - | - | - |
| Lung disease | - | - | 1.53 (1.06-2.21) | - |
| Hypertension | - | - | - | - |
| Body mass index categories | - | - | - | - |
| Normal | - | - | - | - |
| Overweight | - | - | - | - |
| Obese | - | - | - | - |
| Dyslipidaemia | - | - | - | 0.59 (0.37-0.93) ǂ |
| Diabetes | - | - | - | - |
| Alcohol drinker | - | - | - | - |
| Physically active | - | - | - | - |
| Anxiety, self-reported | - | - | - | - |
| Depression, self-reported | - | - | - | - |
| Sensitivity analyses | - | - | - | - |
| Child aged <5 years ǂ | - | - | - | 0.41 (0.21-0.80) ǂ |
| Diagnosed psychiatric diseases ¶ | - | - | - | - |
| Substance use, ever | 3.70 (1.62-8.42) ¶ | 3.70 (1.62-8.42) ¶ | - | - |
| Anxiety, ever | - | - | - | - |
| Depression, ever | 0.60 (0.37-0.99) ¶ | 0.60 (0.37-0.99) ¶ | - | - |

Results were expressed as multivariable-adjusted odds ratio (OR) and 95% confidence interval (CI).

-, not retained in the model. ǂ, sensitivity analyses including child aged<5 years. ¶, sensitivity analyses using PsyCoLaus assessment. *, P-value for trend <0.05.
